# Mechanisms underlying CD19-positive ALL relapse after anti-CD19 CAR T cell therapy and associated strategies

**DOI:** 10.1186/s40364-020-00197-1

**Published:** 2020-05-27

**Authors:** Yuru Nie, Weiqing Lu, Daiyu Chen, Huilin Tu, Zhenling Guo, Xuan Zhou, Meifang Li, Sanfang Tu, Yuhua Li

**Affiliations:** 1 Second Clinical Medical College, Southern Medical University, No. 253, Industrial Avenue, Guangzhou, Guangdong Province China; 2 Department of Hematology, Zhujiang Hospital, Southern Medical University, No. 253, Industrial Avenue, Guangzhou, Guangdong Province China

**Keywords:** Chimeric antigen receptor, CAR T cell therapy, Acute lymphocytic leukemia (ALL), Positive relapse, Mechanism, Strategy

## Abstract

Chimeric antigen receptor (CAR) T cell therapy, especially anti-CD19 CAR T cell therapy, has shown remarkable anticancer activity in patients with relapsed/refractory acute lymphoblastic leukemia, demonstrating an inspiring complete remission rate. However, with extension of the follow-up period, the limitations of this therapy have gradually emerged. Patients are at a high risk of early relapse after achieving complete remission. Although there are many studies with a primary focus on the mechanisms underlying CD19^-^ relapse related to immune escape, early CD19^+^ relapse owing to poor in vivo persistence and impaired efficacy accounts for a larger proportion of the high relapse rate. However, the mechanisms underlying CD19^+^ relapse are still poorly understood. Herein, we discuss factors that could become obstacles to improved persistence and efficacy of CAR T cells during production, preinfusion processing, and in vivo interactions in detail. Furthermore, we propose potential strategies to overcome these barriers to achieve a reduced CD19^+^ relapse rate and produce prolonged survival in patients after CAR T cell therapy.

## Introduction

Chimeric antigen receptor (CAR) T cell therapy has shown revolutionary success in the field of antitumor immunotherapy [1], especially in the treatment for B cell malignancies [2, 3]. Following the first success achieved in a child with acute lymphoblastic leukemia (ALL) after infusion of anti-CD19 CAR (CD19 CAR) T cells in April 201 2[4, 5], several research institutes worldwide have reported CD19 CAR T cell therapy to be a safe and promising treatment for patients with ALL [6, 7] . In total, 67%-85% of patients with ALL receiving CD19 CAR T cell therapy achieve complete remission with a negative minimal residual disease (MRD) status [8–11].

**Fig. 1:**
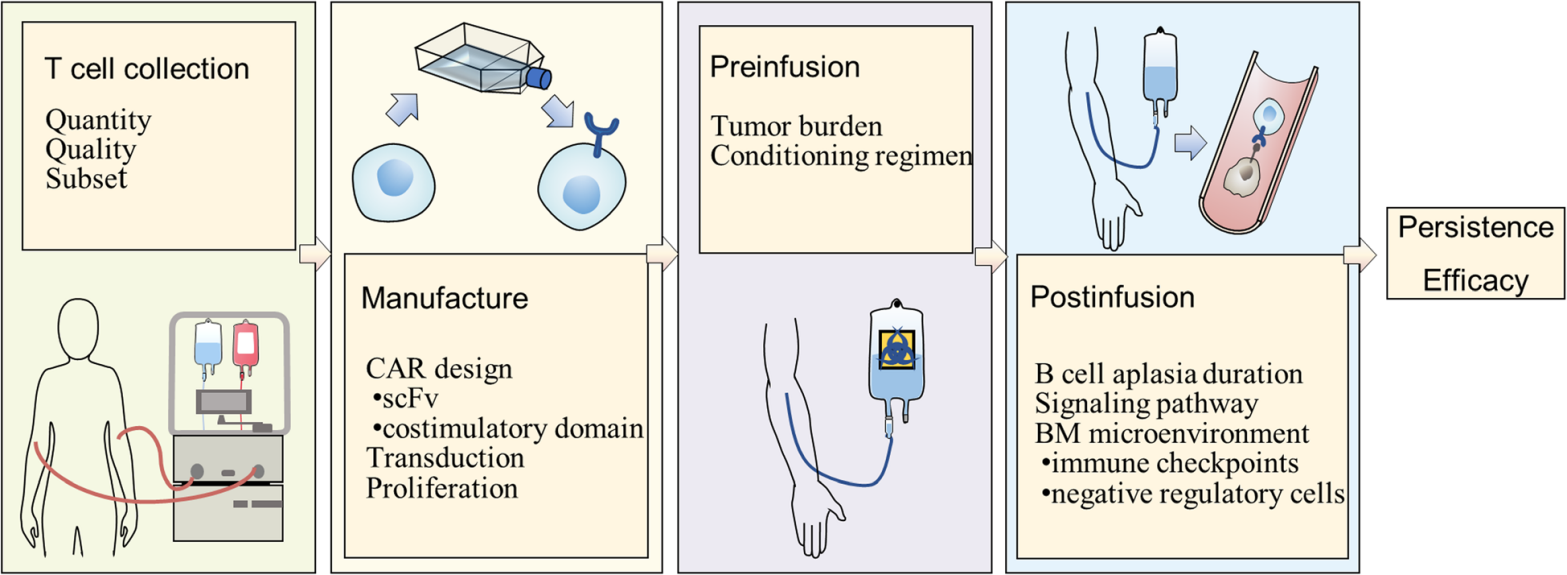
Factors influencing CD19 CAR T cell therapy. The limited persistence and impaired efficacy of CAR T cells could be possible mechanisms underlying CD19^+^ relapse. This figure summarizes potential obstacles to durable remission and better CAR T cell efficacy. First, T cell collection: T cells selected for manufacturing should be of sufficient quantity and good quality and have a phenotype with memory characteristics. Second, CAR T cell manufacture: transgene rejection induced by a murine scFv results in transient in vivo persistence. Selection of the costimulatory domain, transduction technique, especially vector selection, and proliferation method also plays roles in persistence and efficacy. Third, preinfusion: the tumor burden before infusion is associated with patient long-term survival. In addition to lymphodepleting therapy, a conditioning regimen with fludarabine ameliorates T cell persistence. Finally, postinfusion: normal B cells are supposed to recover, but transient B cell aplasia may result in CD19^+^ relapse. Aberrant signaling pathways and the BM microenvironment will impair a T cell’s potential along with its in vivo persistence

However, as more long-term follow-up data are published, a high risk of relapse after CD19 CAR T cell therapy has emerged as a nonnegligible obstacle on the road to improved efficacy and long-term survival. The relapse rate within one year could be even higher than 50%, which indicates a large problem to be solved [12]. To date, there have been studies addressing the mechanism of resistance to CAR T cell therapy with a primary focus on issues related to CD19-negative (CD19^-^) relapse, such as immune escape or antigen loss [13–15]. However, the CD19-positive (CD19^+^) relapse rate following CD19 CAR T cell therapy is higher than the CD19^-^ relapse rate in many trials [7, 16, 17], which can be up to 47.7 %[12]. Barriers to CAR T cell activation and expansion, limited in vivo persistence, and aberrant antileukemia activity are associated with an increased risk of CD19^+^ relapse (Fig. 1). Nonetheless, the mechanisms underlying CD19^+^ relapse are still poorly elucidated.

In this review, we discuss the clinical status of CD19 CAR T cell therapy for ALL, analyzing possible clinical factors for CD19^+^ relapse prediction and/or intervention. Furthermore, we summarize knowledge related to mechanisms underlying CD19^+^ relapse in detail and propose feasible strategies to overcome barriers to durable remission.

## Clinical analysis of CD19-positive ALL relapse after CD19 CAR T cell therapy

### Importance of CAR T cell persistence

A lack of in vivo CD19 CAR T cell persistence is an important causative factor of CD19^+^ relapse after CAR T cell therapy for ALL [18]. Turtle CJ et al. found that CD19^+^ recurrence occurred exclusively in patients without persistent CAR T cells [17]. Three patients were observed to have CD19^+^ relapse after early loss of CAR T cells, while another three patients whose CAR T cells remained experienced CD19^-^ recurrences [11]. The long-term survival of CAR T cells enables continuous surveillance and ongoing clearance of CD19^+^ leukemia cells. Once the CAR T cell frequency diminishes to an undetectable level, abnormal CD19^+^ B cells are likely to repopulate, resulting in antigen-positive relapse.

### Duration of B cell aplasia

Early CD19^+^ relapse is associated with not only limited CAR T persistence but also transient B cell aplasia [6]. Actually, the relatively high expansion peak and prolonged duration of CAR T cells account for delayed B cell recovery in the context of a relatively strong and prolonged cytokine-mediated toxicity profile [19]. According to Maude SL and colleagues’ trial, relapse was heralded by recovery of CD19^+^ B cells in two patients who experienced CD19 CAR T cell loss, while nineteen patients with longer remission were observed to have a longer duration of B cell aplasia [11]. Qian C also found that six subjects developed CD19^+^ relapse following B cell recovery at 2 to 4 months after infusion [20]. Therefore, a relatively limited duration of B cell aplasia is related to an increased CD19^+^ relapse risk, suggesting that monitoring for the return of B cells will be a useful method for predicting ALL relapse and providing a window for consolidation treatment [7, 11].

### Selection of the costimulatory domain

It has been revealed that CAR T cells with 4-1BB as a costimulatory domain (CD19-BBζ) have better persistence than those with CD28 as a costimulatory domain (CD19-28ζ) [21, 22]. Whether the same result is found in clinical practice remains unclear. In current clinical trials of CD19 CAR T cell therapy for ALL, CD19-28ζ CAR T cells can exist in vivo for a median time of 3 months , with almost 7 months as the longest duration [23], while CD19-BBζ CAR T cells have a persistence of up to 20 months [6] and 68% in vivo survival over 6 months in patients [11]. The median CD19^+^ relapse rates of CD19-BBζ and CD19-28ζ CAR T cells are 16.7% and 22.7%, respectively, and the maximums are 31.1% and 47.7% (See Table 1). It appears that patients infused with CD19-BBζ CAR T cells have better CAR T cell persistence and a lower CD19^+^ relapse rate than those infused with CD19-28ζ CAR T cells. However, the trials studying these cells have all been single-armed trials, which lack credibility and statistical significance in comparisons.

**Table 1:** Clinical outcomes after CD19 CAR T cell therapy in patients with ALL. Data is collected from clinical trials for CD19 CAR T therapy in patients with ALL, primarily showing trials’ CAR T design and their outcomes including response rate, relapse rate and so on

study(reference)	CAR design	vector	patient population	conditioning regimen	CAR T cell dose	response,CR persistence	CAR T cell persistence	relapse rate	B cell aplasia time	CRS
Cruz CR et al. [24]	FMC63-28ζ	gammaretrovirus	N=4, peds and adults, 18 y (9-40 y)	none	1.5×10^7^-1.2×10^8^/m^2^,dose escalation	CR: 75% (3/4),3 mo (2-8 mo)	8 w-12 w+	overall: 33.3% (1/3);CD19^+^: 33.3% (1/3)	N/A	none
Lee DW et al. [9]	FMC63-28ζ	gammaretrovirus	N=20, peds and young adults, 15 y (5-27 y)	FC	(0.03-3)×10^6^/kg,dose escalation	CR: 70% (14/20),N/A	detectable up to 68 d	overall: 14.3% (2/14);CD19^-^: 14.3% (2/14)	14-28 d	28.6% (6/31) with Gr 3/4 CRS
Kebriaei P et al. [23]	FMC63-28ζ	sleeping beauty	N=17, adults31 y (21-56 y)	N/A	10^6^-10^8^/m^2^,dose escalation	CR: 64.7% (11/17), 6 mo (2-18 mo)	autologous: 201 d;allogeneic: 51 d	overall: 45.5% (5/11);CD19 expression not tested	N/A	none
Jacoby E et al. [16]	FMC63-28ζ	gammaretrovirus	N=20, peds and adults, 11 y (5-48 y)	FC	1 × 10^6^/kg	CR: 90% (18/20), 28 d-21 mo,1-year EFS and OS rates were 73% and 90%, respectively	median: 23 d	overall: 22.2% (4/18);CD19^+^: 16.6% (3/18);CD19^-^: 5.6% (1/18)	N/A	20% (4/20) with Gr 2/3 CRS
Park JH et al. [12]	SJ25-28ζ	gammaretrovirus	N=53, adults, 44 y (23-74 y)	Cy	(1.5-3)×10^6^/kg	CR: 83% (44/45),N/A, median EFS: 6.1 mo,median OS: 12.9 mo	14 d (7 d-138 d)	overall: 56.8% (25/44);CD19^+^: 47.7% (21/44);CD19^-^: 9.1% (4/44)	N/A	26% (14/53) with sCRS
Tu S et al. [25]	FMC63-28ζ	lentivirus	N=25, adults, 36 y (18-67 y)	FC	median dose of 7.133×10^5^/kg	CR: 88% (22/25),N/A,median DFS: 257 d,median OS: 267 d	up to 11 mo in one patient	overall: 31.8% (7/22);CD19^+^: 22.7% (5/22);CD19^-^: 9.1% (2/22)	N/A	no sCRS
Maude SL et al. [11]	FMC63-BBζ	lentivirus	N=30, peds (n=25),11 y (5-22 y); adults (n=5),47 y (26-60 y)	none/FC/Cy/CAVD/clofarabine/Cy+VP	(0.76-20.6)×10^6^/kg	CR: 90% (27/30), 6 w-8.5 mo, up to 24 mo,6-month EFS: 67%	at 6 months, the probability of CAR T cell persistence was 68%	overall: 29.2% (7/27);CD19^+^: 14.8% (4/27), with early loss of CAR T cells;CD19^-^: 11.1% (3/27)	2-3 mo	27% (8/30) with sCRS
Hu, Y et al. [26]	FMC63-BBζ	lentivirus	N=15, adults<60 y	FC	(1.1-9.8) × 10^6^/kg,dose-escalation	CR: 80% (12/15),1 mo,median LFS: 143 d	up to 7 mo	overall: 50% (6/12);CD19^+^: 16.7% (2/12);both CD19^-^ and CD19^+^: 16.7% (2/12);CNS relapse: 16.7% (2/12)	N/A	40% (6/15) with Gr3 CRS
Gardner RA et al. [7]	FMC63-BBζ	lentivirus	N=43, peds and young adults, 12.3 y (1.3-25.4 y),4 of whom were <3 y	14 given FC	1×10^6^/kg,CD4^+^:CD8^+^=1:1	CR: 93% (40/43), 12.2 mo (1.9-21.5 mo),CR=100% (14/14) in patients after FC,N/A	N/A	overall: 45% (18/40);CD19^+^: 27.5% (11/40);CD19^-^: 17.5% (7/40)	median: 3 mo,time from loss of BCA to relapse: 3.7 mo (0-11 mo)	23% (10/40) with sCRS
Maude SL et al. [6]	FMC63-BBζ	lentivirus	N=75, peds and young adults	72 given FC, 1 given EA	(0.2-4.5)×10^6^/kg	CR: 83% (61/75), 3 mo,EFS and OS rates were 73% and 90% at 6 mo and 50% and 76% at 12 mo, respectively	168 d (20-617 d),as long as 20 mo	overall: 36.1% (22/61);CD19^+^: 1.6% (1/61);CD19^-^: 24.6% (15/61) (3 with concomitant CD19^+^ blasts);unknown: 9.8% (6/61)	probability of BCA at 6 mo after infusion was 83%	73% (55/75) with Gr 3/4 CRS
Jiang H et al. [27]	FMC63-BBζ	lentivirus	N=53, peds and adultsrange, 10-61 y (children: 7.5%; young adults: 54.7%; adult: 37.7%)	FC	(0.89-4.01)×10^6^/kg,CD4^+^:CD8^+^=1:1	CR:88.7%(47/53), 3.4mo,(1.1-15.6mo),median OS: 16.1 mo	up to 18 mo	overall: 44.7% (21/47);2 patients with CD19^+^ relapse at 10 mo and 16 mo;unknown: 40.4% (19/47)	N/A	35.8% (19/53) with Gr 3/4 CRS
Hay KA et al. [28]	FMC63-BBζ	lentivirus	N=53, adults, 39 y (20-76 y)	FC/Cy	2×10^6^/kg,CD4^+^: CD8^+^ =1:1	CR: 85% (45/53), 3.5mo(1.1-17mo),median EFS: 7.6 mo	N/A	overall: 48.9% (22/45); CD19^+^: 31.1% (14/45), 5 with diminished expression;CD19^-^: 13.3% (6/45);unknown: 4.5% (2/45)	CD19^-^ relapse group: ongoing;CD19^+^relapse: 89 d (28-184d)	19% (10/53) with CRS ≥Gr 3
Cheng Z et al. [29]	FMC63-BBζ and FMC63-28ζ	gammaretrovirus	N=6, peds and adults, 26 y (7-45 y)	FC	1 × 10^6^/kg (mixture of 28ζ CAR T cells and BBζ CAR T cells at a 1:1 ratio)	CR: 57.1% (4/7), 9 mo, (2 mo-18 mo),median OS: 12 mo	N/A	overall: 75% (3/4);CD19^+^: 50% (2/4);CD19^-^ : 25% (1/4)	N/A	66.7% (4/6) with Gr 3/4 CRS
Li S et al. [20]	FMC63-BBζ and FMC63-28ζ	lentivirus	N=10, adults, 5 for CD19-28ζ T cells, 5 for CD19-BBζ T cells, 33 y (18-59 y)	FC	(0.1-9.79)×10^6^/kg	CD28 group:CR: 60% (3/5), 4, 6, 8 mo;4-1BB group:CR: 60% (3/5),2 and 8 mo, and 1 is still alive	no more than 1 mo in most patients;persistence over 2 mo was observed only for a patient in the 4-1BB group	CD28 group: 3/3 CD19^+^ relapse;4-1BB group: 2/3 CD19^+^ relapse	2–4 mo in 6 responders	no adverse events were over Gr 2
Cao J et al. [30]	humanized scFv-BBζ	lentivirus	N=18, peds and adults (with or without prior mCAR T cell therapy), 14 y (3-57 y)	FC	1 × 10^6^/kg	CR: 77.8% (14/18), 125 d (100-205 d), OS and LFS rates at d 180 were 65.8% and 71.4%, respectively	median: 60 d, up to 1 y	overall: 28.6% (4/14); CD19^+^: 21.4% (3/14);both CD19^+^ and CD19^-^: 7.2% (1/14)	median 111 d	22.2% (4/18) with CRS≥Gr 3
Zhao Y et al. [31]	humanized scFv-BBζ	lentivirus	N=5, peds and young adults (with prior mCAR T cell therapy), 14 y (9-21 y)	FC	(0.3-3) × 10^6^/kg	CR: 80% (4/5),N/A	30 d-11 mo	N/A	20 d-2 mo	none with CRS>Gr2

Cheng Z et al. performed a study to observe the differences in expansion kinetics, persistence and clinical effects between two kinds of CAR T cells in the same patient. The results showed no significant differences but varied significantly among different patients, suggesting the need for a patient-specific CAR construction design [24] . Qian C et al also carried out a trial to compare these two CAR design options and showed no significant differences. However, positive results were achieved in their subsequent study after they modified the manufacturing method [20]. Therefore, we should treat the problem of costimulatory domain selection relatively cautiously and still need additional randomized controlled clinical trials for verification.

### Consideration of the tumor burden

The tumor burden prior to conditioning regimen administration cannot be excluded from consideration in the clinical application of CAR T cell therapy and plays a role in the relapse of CD19^+^ disease. When stimulated by an increased level of target cells, T cells are more rapidly cleared from the circulation and more likely to be inactivated [9, 25]. Furthermore, overexposure to CD19^+^ target cells can rapidly internalize the CAR on the surface of CAR T cells, which results in damage to the antitumor effect of the CAR T cells [26]. Park J H et al. demonstrated that the preinfusion leukemia load was a good indicator to predict long-term remission duration and the survival rate [12] . The tumor burden was negatively correlated with CAR T cell persistence [12, 25]. It is relatively clear that patients with relatively high levels of leukemia blasts are prone to developing more severe cytokine release syndrome (CRS), for which high-dose steroid therapy is used [12, 18, 27]. However, as cytotoxic drugs, steroids presumably have a negative impact on T cell potential. During a clinical trial, three patients experienced CD19^+^ relapse after administration of high-dose steroids because of their severe CRS resulting from their relatively high leukemia burdens [8, 19]. Thus, tumor load reduction helps to avoid overloaded cytokine storm and induce long-term durable remission. Nevertheless, to produce necessary antitumor efficacy, CAR T cells still require a certain number of target cells for activation and expansion [23]. The balance among the tumor load, CRS, T cell potency and persistence should be seriously considered and controlled.

### Anti-CD19 CAR T cell therapy for different subtypes of ALL

Since there are different ALL subtypes with typical cytogenetic signature respectively, CAR T cell therapy may show different outcomes in patients with certain subtypes. Philadelphia chromosome-positive (Ph+) ALL which associated with aggressive clinical outcome and poor prognosis accounts for approximately 30% of cases of adult ALL [28]. In Davila, M. L.’s trial, CAR T cell therapy was as effective in the 4 Ph+ patients as in the others with relapsed CD19+ ALL after hematopoietic stem cell transplantation (HSCT )[8]. According to another trial in which authors compared outcomes of anti-CD19 CAR T cell therapy between ALL patients with and without Ph+ status, confidence intervals for the differential response rates showed that observed proportion of patients with Ph+ status appeared to be higher than that of patients without Ph+ status, even though these results did not reach statistical significance. However, Ph+ status was proved to have nothing to do with the overall survival in this trial [12].

Genome-wide and other molecular techniques have resulted in the identification of a distinct group of patients with a previously unknown subtype: Philadelphia-like (Ph-like) ALL, which has higher frequency and shorter 5-year survival [28]. Although this genotype is Ph-negative, it has a similar gene expression profile to that of Ph+ ALL but embraces a highly diverse range of genetic alterations activating kinase signaling [29]. Since Ph-like ALL has aberrations affecting critical pathways such as Ras, JAK-STAT [30], and PI3K/AKT/mTOR(6), CAR T cells’ efficacy may be impaired after infusion which will induce a higher risk of disease relapse [32, 33]. Hypodiploid ALL, another subtype, was also recently proved to show activation of Ras and PI3K pathways [34]. However, preclinical and clinical studies concerning CAR T cell therapy for these subtypes are still lacking. Whether ALL patients of different subtypes treated by CAR T cell therapy will achieve consistent remission rate and duration still remain to be determined conclusively.

### Predictive factors for positive relapse in clinical practice

During clinical practice, some factors can be used to predict long-term survival and the possibility of relapse. An elevated prelymphodepletion platelet count, a reduced prelymphodepletion lactate dehydrogenase concentration, a lymphodepletion regimen combined with fludarabine, and no evidence of extramedullary disease or high-risk cytogenetics are associated with better event-free survival. Patients with a relatively high frequency of marrow blasts, decreased persistence of B cell aplasia, or changes in distinct serum cytokines are at an elevated risk of CD19^+^ relapse [7, 9, 35, 36]. Age, performance status, previous allogeneic hematopoietic stem cell transplantation (HSCT) or blinatumomab treatment and the dose of infused CAR T cells did not show any significant effects on long-term survival [23, 35]. However, in a clinical trial carried out in our department, we found that elderly patients were at an increased risk of disease relapse or progression, which requires a further study to confirm the relation between age and relapse risk [37].

## Mechanism of CD19^+^ relapse

### Manufacture of CAR T cells in vitro

#### T cells are insufficient in quantity and poor in quality

To manufacture CAR T cells requires collecting enough good-quality T cells. Due to previous cytotoxic therapy, patients often develop lymphopenia, preventing the harvest of adequate amounts of T cells for CAR T cell therapy. Furthermore, it has been observed that chemotherapeutic regimens containing clofarabine or doxorubicin are associated with an insufficient quantity or poor quality of CAR T cell products [9]. Moreover, clinical data suggest that previous treatment with cyclophosphamide and cytarabine can selectively reduce the frequency of early-lineage T cells associated with CAR T cell expansion [38].

Prior treatments before CAR T cell therapy, such as chemotherapy, can affect the quality of the T cells collected. Multiple chemotherapies affect the metabolic pathways of T cells in vivo, thereby affecting the quality and persistence of CAR T cells [39] .

#### CAR T cell products generated from different T cell subsets have different potencies

At present, the T cell subsets used for CAR T cell manufacture are not strictly defined [40], so CAR T cell products are composed of CD4^+^ and CD8^+^ T cells with an activated effector T (T_E_) cell phenotype [2]. Naïve T (T_N_) cells can differentiate into T memory stem (T_SCM_) cells, central memory T (T_CM_) cells and effector memory T (T_EM_) cells, with the lifespan gradually shortening and the self-renewal ability weakening [40, 41]. However, T_EM_ and T_E_ cells used for adoptive immunotherapy have poor proliferative and survival abilities, which can lead to relapse [41].

#### Construction of a CAR

##### Murine single-chain variable fragments (scFvs) induce an immune response

At present, most CAR T cell products, especially scFvs, used in clinical trials are derived from mice (FMC63 [42] or SJ25C1-mAb), which could induce immunological rejection in vivo, particularly via CD8^+^ T cell-mediated immune responses [43]. In a clinical trial, Cameron J. Turtle et al .[17] found that there was no expansion or persistence of CAR T cells in 5 of 6 patients with CD19^+^ relapse when the authors reinfused the products after relapse and an immune response to the CAR transgene product was found in all of the patients. When CAR T cells were used for other solid tumors, there were different degrees of immune rejection as well [44–47]. Moreover, tonic signaling from murine CAR T cell clusters of CAR molecules results in the exhaustion of CAR T cells [17]. Therefore, reinfusion of CAR T cells into patients with CD19^+^ relapse after mouse CAR T cell infusion is generally ineffective.

##### Selection of a costimulatory domain

**Fig. 2:**
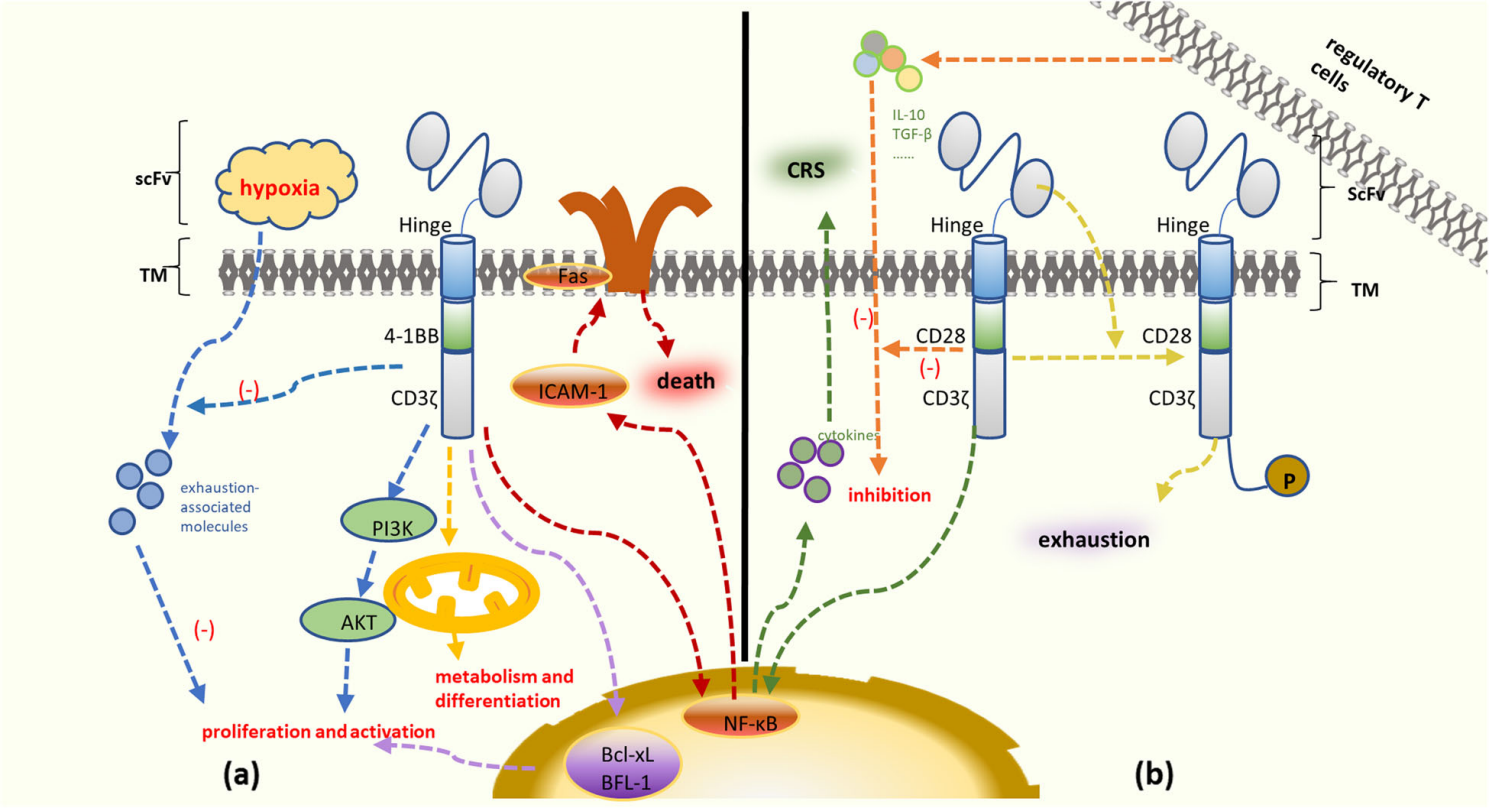
Main signaling pathways involved in CD19-BBζ T cells and CD19-28ζ T cells. **a** A high mitochondrial respiratory capacity promotes metabolism and differentiation. 4-1BB domain signaling activates the PI3K pathway and upregulates Bcl-xL and BFL-1 expression. Tonic CAR-derived 4-1BB signaling activates the NF-B pathway and enhances FAS-dependent apoptosis. CD19-BBζ T cells diminish the expression of exhaustion-associated molecules more than CD19-28ζ T cells. **b** The main signaling pathways involved in CD19-28ζ T cells. CAR T cell inhibition induced by regulatory T cells, IL-10 and TGF-β can be reduced by the incorporation of the CD28 domain. CD19-28ζ T cells exhibit enhanced activation of the transcription factor NF-B and promote cytokine secretion. CD19-28ζ T cells are more likely to result in the development of severe CRS than CD19-BBζ T cells. However, tonic CAR CD3ζ phosphorylation triggered by clustering of the CAR single-chain variable fragment (scFv) leads to more rapid exhaustion

Second-generation CAR T cells containing a CD3ζ signaling domain coupled with another costimulatory molecule exhibit improved proliferation and function. CD28 and 4-1BB are the two costimulatory domain options used in most preclinical and clinical studies. 1. CAR T cell inhibition induced by T regulatory cells (Tregs) and the immunosuppressive molecules IL-10 and TGF-β can be reduced by the corporation of the CD28 domain [48]. 2. CD19-28ζ T cells are also able to induce an augmented antitumor response and increased proliferation via enhanced activation of the transcription factor NF-κB, which helps to promote cytokine gene transcription and secretion [49]. 3. It has been confirmed that CD19-28ζ T cells show a strong functional potential and high expansion but expansion was subsequently followed by relatively rapid exhaustion after tonic CAR CD3ζ phosphorylation triggered by clustering of a CAR scFv [21]. Since CD19-28ζ T cells produce a robust antileukemia response and exhibit increased cytokine secretion, patients infused with these cells are relatively likely to develop severe CRS and need steroid therapy. However, high doses of lymphotoxic steroids limit the proliferation and persistence of CD19-28z CAR T cells and lead to recurrence while tocilizumab does not, which the specific mechanism remains unknown [8, 19]. Therefore, CD19-28ζ T cells can mediate rapid remission, but the risk of relapse will be high, remaining unfavorable for long-term survival (Fig. 2a)

CD19-BBζ T cells have a high mitochondrial respiration capacity, providing increased oxidative metabolism and differentiation into central memory T (T_CM_) cells [50, 51]. 4-1BB domain signaling can also activate the AKT/mammalian target of rapamycin pathway and upregulate the expression of the antiapoptotic genes Bcl-xL and BFL-1, which is beneficial to T cell survival and proliferation [22]. In contrast to CD19-28ζ T cells, CD19-BBζ T cells have better persistence with diminished expression of exhaustion-associated molecules obtained by a stronger response to hypoxia, upregulated cellular metabolism and negative regulation of apoptosis [21].

Although CD19-BBζ T cells are related to prolonged persistence, their duration in vivo is still limited because of certain mechanisms of self-death. When using a gamma retroviral vector, tonic CAR-derived 4-1BB signaling persistently activates the NF-κB pathway via tumor necrosis factor receptor-associated factor 2 (TRAF2), which upregulates the expression of intracellular adhesion molecule 1 (ICAM-1) and enhances FAS-dependent apoptosis [52, 53] (Fig. 2b)

In addition to CD28 and 4-1BB, other costimulatory domains such as OX40 and ICOS also have potential to induce persistence and antitumor responses, which has been revealed in preclinical studies [54, 55]. Moreover, Toll-like receptors (TLRs) play an important role in the immune response. It has been showed that after induction chemotherapy, the mRNA expression of TLR2 was significantly lower as compared to before treatment [56]. There are many other costimulatory domains, such as Dap10 costimulatory domains, 2B4 costimulatory domains, GITR domains, CD150 signaling lymphocytic activation molecule (SLAM), a 150-kDa protein termed as M150. Different costimulatory domain induces differential effects in CAR-expressing T cells [57–61].

#### Expansion of CAR T cells in vitro

Matthew E. Pipkin et al. suggested that strong IL-2 signaling had a negative impact on CD8^+^ T cell memory formation but did not influence secondary expansion. IL-2 could also promote effector cytotoxic T lymphocyte (CTL) differentiation and senescence [62]. A meta-regression analysis article analyzed the relationship between “adding IL-2 during cell culture” and the overall response rate (ORR) of cultured CAR T cells. The results of the analysis showed that the cells cultured without the addition of IL-2 had a higher ORR than those cultured with IL-2 [63]. The ORR of CAR T cells can result in a worse effect, which leads to relapse.

#### Transfection can lead to CAR T cell recurrence

Virus-based vectors are the most commonly used gene delivery system. Studies have shown that retrovirus transfection of T cells introduces vector-derived immunogenic epitopes into patient’ T cells. Therefore, anti-CAR T cells produced in patients target both the CAR vector and retroviral vector epitopes. As a result, CAR T cells are likely to be identified and attacked in vivo, CAR T cell persistence is reduced, and recurrence occurs [45].

Another important side effect of CD19 CAR T cell therapy is B cell hypoplasia. Due to the integrability of lentiviral vectors (LVs) and the persistence of T cell clones after adoptive transfer, LV-modified T cells appear to be able to persist for >5 years after treatment and continue to induce B cell hypoplasia, which may lead to recurrence [64].

Virus-based vectors also have other potential safety problems. They can integrate semirandomly into the human genome, which can lead to insertional mutagenesis and make the engineered cells become tumorigenic [65] .

In recent years, the most widely used transposon is the sleeping beauty (SB) transposon system. Although the efficiency of SB-mediated stable gene transfer is significantly higher than that of conventional DNA-mediated random integration, because SB inserts randomly, there are still many questions about safety in clinical applications [66].

CRISPR/Cas9 technology originates from type II CRISPR/Cas9 systems. However, CRISPR/CAS9 gene editing induces off-target cleavage events in therapeutic applications depending on the experimental environment and cell type. Therefore, the problem we need to consider is to what extent these nucleases induce off-target cleavage events [67].

## Aberrant signal transduction pathways

The limited in vivo persistence, cytotoxicity and expansion of CAR T cells could also be due to aberrant signaling pathways within CAR T cells or interactions between CAR T cells and tumor cells.

BCR-activated signal transduction pathways, such as the PI3K/AKT/mTOR and JAK2/STAT signaling pathways, are related to CAR T cell potency. Zheng W et al. demonstrated that tonic signaling through CAR CD3ζ ITAMs led to activation of PI3K signaling and was associated with a more differentiated phenotype [32]. In contrast, T cells with a less differentiated phenotype, such as TCM and TSCM cells, are associated with increased persistence and improved antitumor reactivity and expansion. Urak R and colleagues also described the correlations between PI3K/AKT/mTOR signaling inhibition and CAR T cell populations with memory-like characteristics that have prolonged persistence and comparatively high antitumor activity [68]. In addition, JAK2 genetic mutation has been observed in many hematological malignancies. IL-6/JAK/STAT3 signaling acts to drive the proliferation, survival, invasiveness, and metastasis of tumor cells, inducing upregulation of the expression of the immune checkpoint molecules PD-1 and/or PD-L1 and thus suppressing the immune response, which impairs the function of CAR T cells [33].

Abnormalities in the apoptotic pathway employed by CD19 CAR T cells are thought to be another mechanism underlying impaired CAR T cell cytotoxicity. Tumor necrosis factor-related apoptosis-inducing ligand (TRAIL) apoptotic pathway-mediated killing is the principle pathway through which CD19 CAR T cells induce tumor cell apoptosis [69]. Cross-resistance to TRAIL results from downregulated expression of death receptor-5 (DR-5), which leads to dysfunction in CD19 CAR T cells [70].

The cGAS-STING signaling pathway can be activated by dsDNA released during tumor cell division. Downregulation of cGAS-STING signaling leads to less type I interferon (IFN), which plays important roles in the antitumor response and prevention of T cell apoptosis [71]. Moreover, tumor cells can produce adenosine to suppress the antitumor ability of CD19CAR T cells via the adenosine receptor 2A signaling (A2Ars) pathway [72].

## Bone marrow (BM) microenvironment

**Fig. 3:**
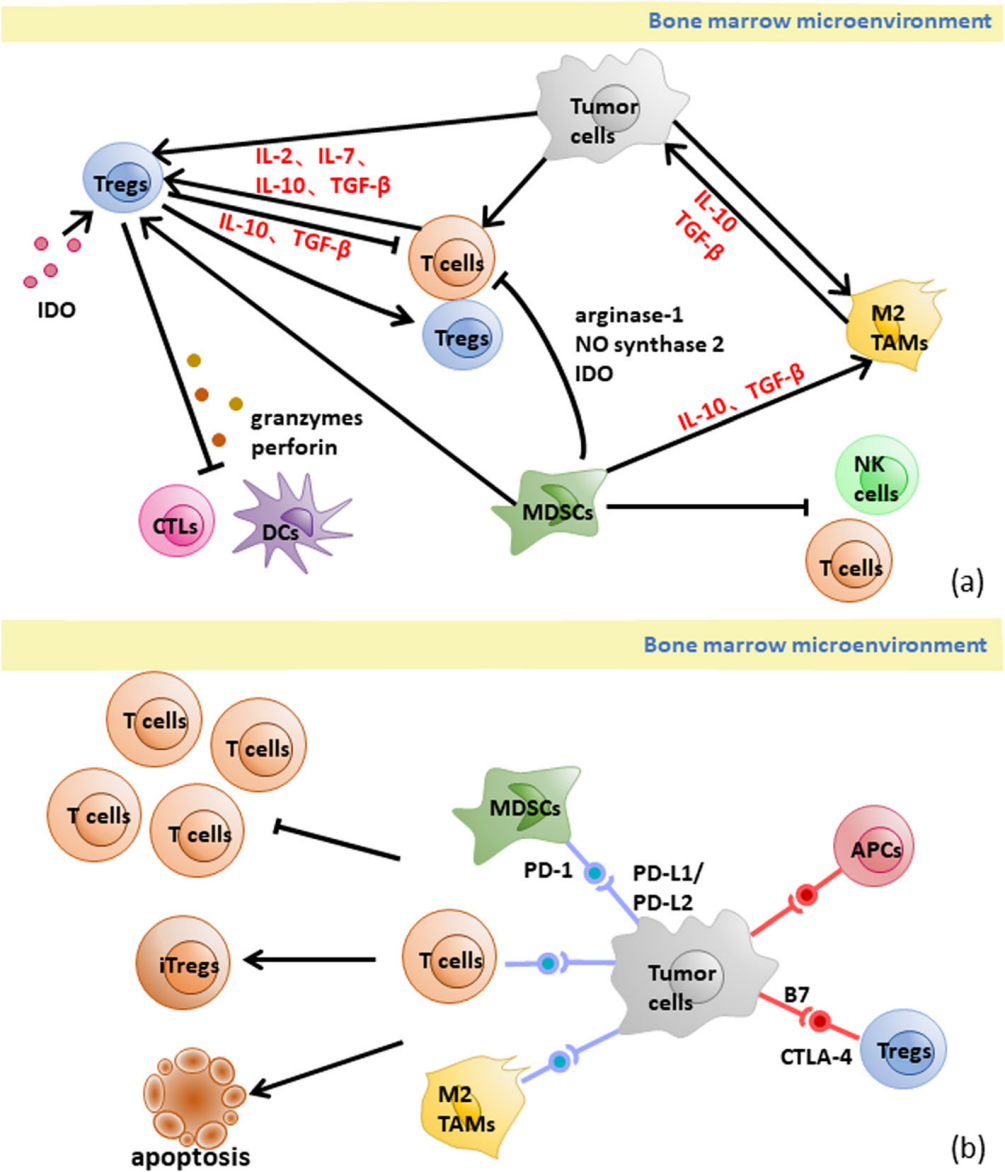
The mechanism of CD19+ relapse in BM microenvironment. **a** Main interaction between negative regulatory cells, tumor cells and immune effector cells in BM microenvironment. Tregs, MDSCs and TAMs suppress CTLs, DCs, NK cells and T cells by cytokines, enzymes and cell-cell interactions. Negative regulatory cells and tumor cells attract and improve each other’s recruitment, differentiation and expansion. Tregs: regulatory T cells; MDSCs: myeloid-derived suppressor cells; TAMs: tumor associated macrophages; IDO: indoleamine-2, 3-dioxygenase; CTLs: cytotoxic T cells; DCs: dendritic cells; NK cells: natural kill cells; TGF-β: transforming growth factor β. **b** The negative regulation checkpoint in BM microenvironment. The PD-1/PD-L1 pathway between tumor cells and MDSCs, T cells, TAMs inhibits the proliferating of T cells and transforms T cells into induces Tregs or induces apoptosis. The CTLA-4/B7 pathway suppresses APCs while activates Tregs. (Tregs: regulatory T cells; iTregs: induced Tregs; TAMs: tumor associated macrophages; APCs: antigen-presenting cells; MDSCs: myeloid-derived suppressor cells; PD-1: programmed death-1; PD-L: programmed cell death 1 ligand)

It has been reported that the tumor microenvironment leads to relapse via immunosuppression, mostly in solid tumors. However, studies on the role of the BM microenvironment in ALL relapse have not been published. Here, we inferred the mechanism of relapse in the BM microenvironment based on other leukemias treated with CAR T cell therapy (Fig. 3).

### Suppressive regulatory cells inhibit the immune function of the BM microenvironment

Tregs inhibit normal immune cells in the tumor microenvironment, allowing tumor cells to exist [73] primarily via four mechanisms: (1) induction of T cell apoptosis via cell-cell interactions; (2) inhibition of immune responses by cytokine secretion; (3) release of perforin and granzymes to kill CTLs, monocytes and DCs directly; and (4) suppression of local immune responses through expression of cytotoxic T-lymphocyte-associated protein 4 (CTLA-4) [74–76]. Furthermore, IL-2, IL-7, IL-10 and TGF-β have been proven to lead to the recruitment, differentiation and expansion of Tregs [75].

It has been observed that myeloid-derived suppressor cells (MDSCs) inhibit human CAR T cell responses in tumors, including the CD19 CAR T cell response in lymphoma [77–79]. MDSCs inhibit T cells via seven mechanisms: (1) inhibition of the proliferation and activation of CD4^+^ and CD8^+^ T lymphocytes through arginase-1 or nitrogen oxide synthase 2 as well as IDO; (2) alteration of the macrophage phenotype toward a type 2 response and associated activity by increasing the IL-10 level; (3) inhibition of the cytotoxicity, natural killer group 2D (NKG2D) expression, and IFN-gamma production of natural killer (NK) cells; (4) induction of Tregs; (5) upregulation of PD-1 expression on MDSCs via hypoxia-inducible factor-1α; (6) deregulation of Fas-mediated apoptosis; and (7) establishment of immunosuppression via the GM-CSF/JAK2/STAT3 pathway [77–82].

Tumor-associated macrophages (TAMs) are recruited by tumor cells via cytokines such as CSF-1 and IL-10 and converted into M2 macrophages [83], which have anti-inflammatory, angiogenic and tumor-promotive effects [74]. TAMs attract chronic lymphocytic leukemia (CLL) cells by secreting CXCL12 and CXCL13 and protect CLL cells from spontaneous or drug-induced apoptosis via CXCL12, BAFF, APRIL, CD31, and Plexin-B1 and activating the BCR signaling cascade [84].

### Suppressive immune checkpoints inhibit the activation of T cells

Like CD28, CTLA-4, which is expressed on antigen-presenting cells (APCs), has B7 ligands (CD80 and CD86), which have been shown to exhibit upregulated expression in acute myelocytic leukemia (AML) cells [85], and the affinity of CTLA-4 for the B7 ligands is higher than that of CD2 8[86]. Once bound to a B7 molecule, CTLA-4 downregulates TCR activation by competing for CD28 and initiating suppressive signals at the same time, which are involved in the negative regulation of the immune response. However, CTLA-4 is constitutively expressed on Tregs and provides activation signals to these cells [81, 85].

It has been reported that the expression of PD-L1 and/or PD-L2 in many AML cells can be further upregulated in the presence of activated T cells, including CAR T cells, mainly through the production of IFN-γ [81, 85]. The PD-1/PD-L1 pathway modulates immunosuppression primarily via the following mechanisms: (1) binding of PD-L1 on the surface of tumor cells, TAMs or MDSCs with PD-1 on the surface of tumor-specific T cells to induce apoptosis and depletion of tumor-infiltrating lymphocytes in the tumor microenvironment; (2) preventing T cells from proliferating by selectively inhibiting the RAS/ MEK/ERK and PI3K/AKT signaling pathways, accordingly blocking cell cycle-related gene transcription and protein expression; and (3) promoting the transformation of CD4^+^ T cells into induced Tregs (iTregs) through PD-L1 expressed on APCs [81].

In addition, IDO is highly expressed in the microenvironments of various tumors and shows high protein expression in the peripheral lymphoid organs (the lymph nodes, spleen and tonsils [87]. Overexpression of IDO promotes immune tolerance mainly through the production of kynurenine, which can inhibit the proliferation and differentiation of CD8^+^ T cells, activate aromatic receptors and promote the differentiation of Tregs, resulting in the inhibition of the antitumor immune response [81, 85, 88, 89].

## Strategies

### Sufficient quantity of high-quality T cells

The manufacturing of a CAR T cell product is feasible with a CD3^+^ cell count of ≥150 cells/μl [14]. Furthermore, collecting T cells from patients at a high risk of recurrence early or before treatment may improve the quality of the produced CAR T cells [14]. To avoid the toxic effects of multiple chemotherapies on cells, we can reduce the frequency of chemotherapy and increase the interval time. Additionally, creating CAR T cell product from healthy donor cells is an alternative strategy to avoid poor CAR T cell quality. Currently, several research groups are testing donor-based CAR T cell therapeutic strategies in the contexts of clinical [90, 91] and posttransplant relapses in patients who can be treated with T cells derived directly from the original allogeneic stem cell donors. The results show only a low frequency of high-grade graft-versus-host disease (GVHD) [92–94].

### Selective manufacture of CAR T cells from the memory stem cell subset

**Fig. 4.**
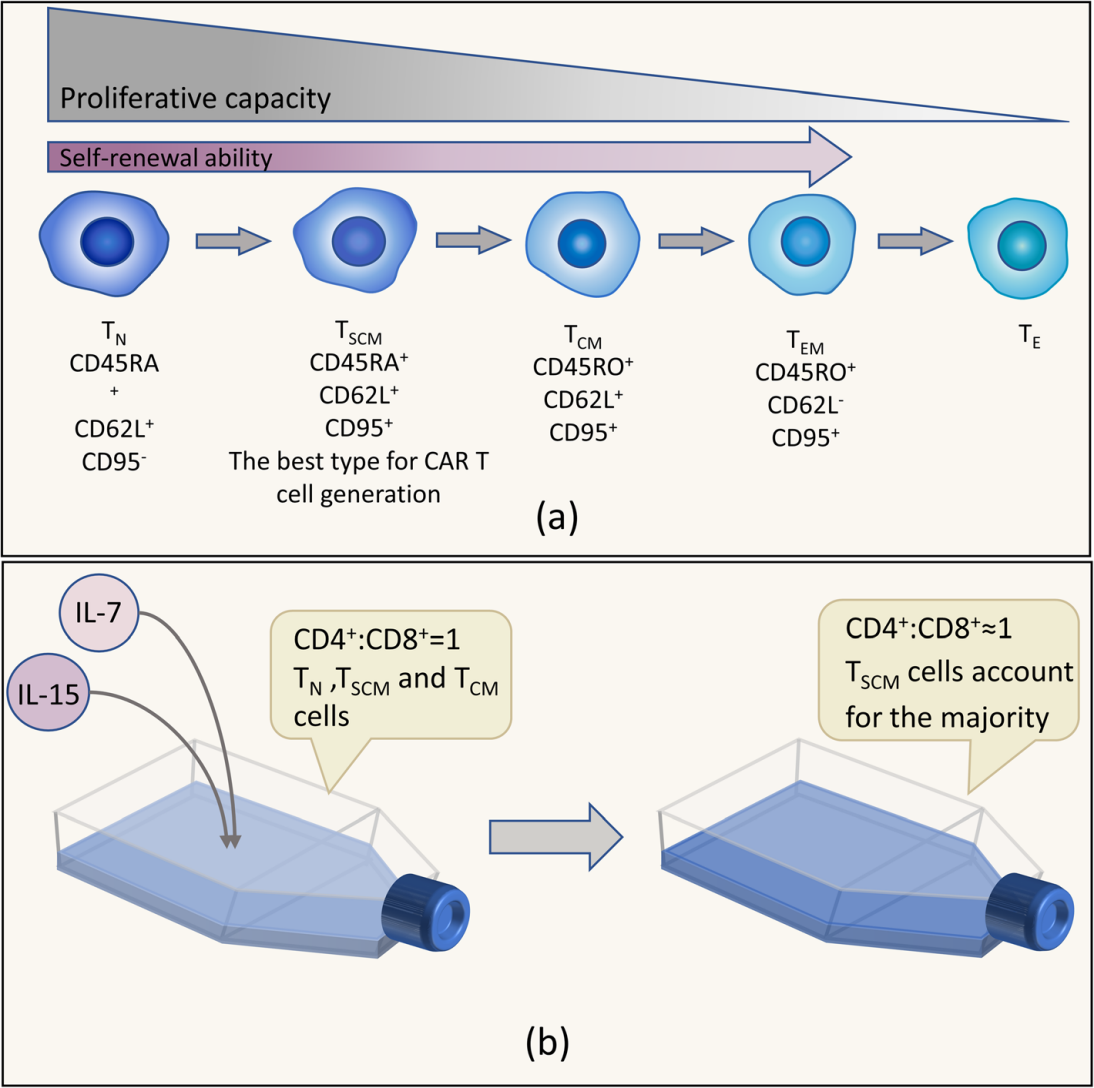
Some key points during the process of manufacturing CAR T cells. **a** The progress of T cells with the associated characteristics. As T_N_ cells differentiate, the proliferative capacity of T cells is gradually reduced. Except for T_E_ cells, the remaining subsets have a self-renewal ability, which declines from T_N_ to T_EM_ cells. Therefore, it is best to choose T_SCM_ cells for CAR T cell generation. **b** Adding IL-7/IL-15 during in vitro expansion has a positive impact. IL-7/IL-15 can increase the proportion of T_SCM_ cells and contribute to maintaining the ratio of CD4^+^:CD8^+^ cells.

Selecting the memory stem cell subset can improve the persistence and efficiency of CAR T cells. T_SCM_ cells defined as CD62L^+^CCR7^+^CD45RA^+^CD95^+^CD45RO^−^CD28^+^CD27^+^IL2Rb^+^IL7Rα^+^ cells not only have stem cell-like properties, including the abilities of self-renewal and multipotency, but can also differentiate into T_CM_ and T_EM_ cells in vitro, which have enhanced properties of self-renewal, proliferation and survival [41, 95] (Fig. 4a). Researchers analyzed the distributions of T cell subsets in the peripheral blood from 92 healthy subjects ranging from 3 to 88 years old and found that T_SCM_ cells accounted for 1.27 ± 0.55% of the CD4^+^ and 0.98 ± 0.53% of the CD8^+^ T cell populations and that the absolute number of CD8^+^ T_SCM_ cells decreased with age, while the number of CD4^+^ T_SCM_ cells was stable [96].

There is an experiment showing that CD19 CAR–modified T_SCM_ cells have enhanced metabolic fitness and mediate robust long-lasting antitumor responses [42]. In a clinical trial by Franziska Blaeschke et al. [97] that included 4 pediatric ALL patients, CAR T cell products consisting mostly of T_CM_ and T_SCM_ cells had excellent proliferative and antitumor capabilities in vivo. Moreover, the products created with the protocol chosen in the trial expressed rather low levels of inhibitory checkpoint molecules on the cell surface, which indicated their persistence and high efficiency [97]. A clinical trial by Joseph A. Fraietta et al. showed that the median frequency of T_SCM_ cells in CAR T cells products was modestly high in complete remission patients [43]. Therefore, selecting a memory stem cell subset is a strategy to enhance the persistence and killing capacity of CAR T cells.

It has been demonstrated that the induction of Wnt/β-catenin signaling using inhibitors of glycogen synthase kinase 3β (GSK-3β), such as TWS119, helps to enrich T_SCM_ cells by inhibiting differentiation in both mice and humans [98, 99]. Karolina Pilipow et al proved that adding antioxidants, especially N-acetylcysteine (NAC), to the manufacturing procedure for CD19 CAR T cells inhibited effector differentiation while enabling expansion of CD8^+^ T_SCM_ cells and stem cell-like CD8^+^ T cells [100]. Therefore, the T_SCM_ cell subset is the best choice for manufacturing CAR T cell products.

### The immune response decreases with the expression of humanized scFv in CAR T cells

In several basic research experiments, humanized CAR T cells were designed and manufactured with optimized murine scFv fragments, and the effects were evaluated in cells or mice. It was demonstrated that humanized CAR T cells exhibit an antitumor ability similar to that of murine CAR T cells with much lower immunogenicity and enhanced persistence [101–103]. Moreover, humanized CAR T cells will not lead to CAR T cell exhaustion because of the lack of tonic signaling and the ability to receive multiple stimuli from CD19^+^ tumor cells [102, 103].

In addition to studying scFvs, a study transformed hinge and transmembrane domains into human-derived versions and compared CARs with CD28 costimulatory moieties along with hinge and transmembrane domains from either the human CD28 molecule or the human CD8α molecule. It has been proven that T cells expressing CARs with the CD8a hinge and transmembrane domains (Hu19-CD828Z) can bear multiple CD19^+^ cell stimulations and exhibit reduced levels of activation-induced cell death (AICD). In addition, the study found that the engineered T cells expressed reduced levels of PD-1 and LAG-3 so these cells could be preserved for a relatively long time. The authors found that T cells expressing Hu19-CD828Z had good antitumor activity with reduced cytokine production [101].

In addition, some clinical studies have confirmed that humanized CAR T cells can produce good antitumor effects on patients, both new patients and highly treated patients [104–106]. In particular, for those patients who experienced disease relapse after murine CAR T cell therapy and failed reinfusion, infusion of humanized CAR T cells can achieve some degree of remission or even a MRD^-^ status [105].

The current limitation is that the generation of a fully human-derived CAR is difficult to accomplish even if all segments of the CAR are humanized. For example, amino acid sequences not naturally expressed in humans may be present at junctions between the different components of the CAR, such as the linker connecting the light chain and heavy chain variable regions, and in the idiotypic epitopes of the variable regions [101]. In this regard, comprehensive testing of CAR constructs with bioinformatic tools to identify potential immunogenic sequences is available for removing or altering specific sites to prevent anti-CAR immune responses [101, 102].

### Selection of the 4-1BB stimulatory domain is optimal

In current clinical trials of CD19 CAR T cell therapy for ALL, CD19-28ζ CAR T cells can exist in vivo for a median time of 3 months, while 68% of patients infused with CD19-BBζ CAR T cells exhibit cell survival for over 6 months [11]. The median CD19^+^ relapse rates of CD19-BBζ and CD19-28ζ are 16.7% and 22.7%, respectively. Studies have shown that CD19-28ζ T cells lead to rapid expansion of CAR T cells in vivo but also to rapid cell depletion and a high recurrence rate. In contrast, CD19-BBζ T cells are characterized by relatively mild in vivo cell expansion but with a longer duration and a lower recurrence rate [21]. Therefore, it would be better to use CD19-BBζ CAR T cells. Furthermore, another study showed that the configuration utilizing two signaling domains (CD28 and CD3ζ) and the 4-1BB ligand provided the highest efficacy, showing balanced tumoricidal function and increased T cell persistence [31]. Therefore, in third-generation CAR T cells, multiple costimulatory receptor domains are added, and CD28 combined with 4-1BB may achieve both durable and powerful effects. A study has showed that the adenine base editor (ABE )editing reduced the expression of PD-1 in CART-T cells by changing the coding sequence of N74 residue of PDCD1 and can enhance cytotoxic functions in vitro and in vivo [107].

### The ratio of CD4^+^:CD8^+^ CAR T cells can impact product efficacy

It is optimal that CAR T cell products consist of CD4^+^:CD8^+^ T cells at a 1:1 ratio. It has been proven that the ratio of CD4^+^:CD8^+^ CAR T cells influences antitumor efficacy and the persistence of CAR T-cell products is best when the products are composed of CD4^+^:CD8^+^ T cells at a 1:1 ratio [17, 40]. It is feasible to sort cells before infusion to ensure that the ratio of CD4^+^:CD8^+^ CAR T cells is 1:1 or expand CAR T cells at a 1:1 ratio while adding IL-7/ IL-15 to maintain the ratio [108].

### During expansion of CAR T cells, addition of IL-7 and IL-15 improves the persistence and activity of CAR T cells

Several in vitro experiments have shown that IL-7 and IL-15 can induce T_N_ cells to expand into memory T cell subsets and, compared with IL-2, these cytokines are beneficial for preserving the phenotype of T_SCM_ cells [108–110]. A clinical trial showed that IL-7 and IL-15 could produce prolonged survival of CAR T cells exposed to repeated serial antigen stimulation [108]. Moreover, it has been demonstrated in mice that CD19 CAR–modified T_SCM_ cells facilitate efficient homing to the secondary lymphoid organs and proliferate continuously in vivo [108, 110]. In an in vitro study, researchers confirmed that a short period of CD3/CD28 costimulation (48 h) increased the percentages of T_SCM_ cells within the CD4^+^ and CD8^+^ subsets when the cells were cultured with IL-7/IL-15 and that adding IL-21 to the culture further increased the number of T_SCM_ cells [111] (Fig. 4b). However, whether IL-7 and IL-21 play a positive role in expansion is controversial. A study by Darya Alizadeh et al demonstrated that IL-15 preserved the T_SCM_ phenotype by reducing mTORC1 activity but the addition of IL-7 and/or IL-21 reduced the benefits of IL-15 on the CAR T cell phenotype and antitumor activity [112]. Thus, this area remains to be further studied. In addition, the ratio of CD4^+^:CD8^+^ CAR T cells can remain stable in culture medium with added IL-7 and IL-1 5[108].

### Using an LV can increase the persistence and expansion of CAR T cells

Lentiviruses can transduce not only dividing cells (without nuclear membranes) but also nondividing cells (with nuclear membranes), whereas retroviruses can only transduce dividing cells [113]. Lentiviral preintegration complexes can enter the nucleus via the ATP-dependent nuclear pore complex. Cells retain greater functional potential when they are transduced in their inactive state than when transduced in their active state [64]. Because of this, LVs will have a higher probability of modifying less differentiated naive T cells than retroviral vectors, which will increase the persistence and chance of survival of the resulting CAR T cells.

Another study proved that the expression of BB.z.CD19 and GD2 CARs from an LV could significantly reduce cell death and restore the overall expansion of CAR T cells in vitro. Therefore, BB.z CD19 CAR T cells produced with LVs could lead to a significant extension in survival [53]. In addition, various clinical trials have proven that compared with that of other viral vectors, the integration pattern of LVs has significantly lower risks of carcinogenic transformation and random transgenic integration [65]. Therefore, it would be better use LVs.

### Increased persistence of CAR T cells mediated by regulating signaling pathways

#### BCR-mediated signaling pathways

The PI3K/AKT/mTOR pathway is a BCR-mediated signaling pathway. Treatment with PI3K inhibitors during CAR T cell in vitro amplification can increase TN/TSCM and TCM cell numbers [32, 68]. In addition, it can not only significantly decrease the expression of PD-1 and Tim-3 but also mediate increased expression of the lymphoid homing marker CD62L [114]. In conclusion, induction with inhibitors during in vitro amplification can improve the persistence, antitumor effect and in vivo amplification of CAR T cells.

#### The TRAIL apoptotic pathway

CD19 CAR Transduced primary human CTLs kill CD19 human non-Hodgkin lymphomas (NHLs) mainly via the TRAIL apoptotic pathway [69]. Because histone deacetylase inhibitors (HDACis) can upregulate the expression of DR5 in the TRAIL pathway, they can sensitize the pathway to promote tumor apoptosis. Furthermore, HDACis also reduce the expression of antiapoptotic proteins such as Bcl [70].

#### The cGAS-STING pathway

In the cGAS-STING pathway, the downstream molecule type I IFN has various immunostimulatory functions, which can improve the cytotoxic effect of T cells or NK cells and prevent apoptosis in effector T cells. Therefore, a cGAS-STING agonist can be used as a sensitizer of CAR T cells [71].

#### The A2ARs pathway

Tumor cells produce adenosine, which inhibits the antitumor ability of endogenous T cells through activation of the A2ARs pathway [72]. Therefore, an A2AR antagonist or shRNA targeting the A2AR gene can be used to reverse this problem and improve CAR T cell efficacy. The effectiveness of this approach can be improved by combination with pd-1 inhibitors [115, 116].

### Strategies focused on the BM microenvironment

Antibodies targeting Tregs, fludarabine and cyclophosphamide can reduce the number of Tregs and augment the efficiency of CAR T cells. Before starting CAR T cell therapy, lymphodepletion in patients using fludarabine and/or cyclophosphamide decreases the number of circulating T cells, including Tregs, and thereby induces the proliferation of transferred T cells by reducing competition for IL-7 and IL-15, which support the proliferation of preexisting T cells [88, 117]. T cells with a modified CD28δ-CD3ζ CAR, which are deficient in CAR-induced IL-2 secretion, reduce Treg numbers [73, 117, 118].

Retinoids and all-trans retinoic acid have been shown to diminish the suppressive effects of MDSCs, and coadministration of these molecules may enhance the efficacy of CAR therapies [77, 119]. Gene-modified NK cells bearing a chimeric receptor with the activating receptor NKG2 can kill autologous intratumoral MDSCs [120].

Suppressing the function of CSF-1 may be a great measure for reducing recurrence as CSF-1 plays a role in recruiting M2 macrophages. Zhang P. et al .[83] designed CAR T and CAR-NK cells targeting CSF1R to block the promotive effect of CSF-1 on M2 macrophages and obtained results indicating efficacy. Also, Antibodies targeting CD47 on the macrophage cell surface can reduce the suppression of BM microenvironment [1].

With the development of knowledge on CTLA-4, antibodies targeting CTLA-4, such as ipilimumab, have been studied in experiments to augment the effect of CAR T cells. The antitumor mechanisms of anti-CTLA-4 antibodies primarily include the following two effects: (1) modulation of tumor-specific immune effector cells, for instance, affecting CD8^+^ T cells to promote clonal proliferation and (2) removal of Tregs to relieve the inhibition of the tumor-directed immune response [81].

Blocking the PD-1/PD-L1 signaling pathway is expected to restore the function of effector CD8^+^ T cells while suppressing the functions of Tregs and MDSCs, accordingly enhancing the antitumor effect of the immune system [81]. In a phase I/II study, relapsed/refractory (RR)-AML patients with poor risk features were treated with the anti-PD-1 antibody nivolumab and azacitidine every 4-5 weeks indefinitely. Eighteen percent of the patients achieved complete remission/complete remission with incomplete blood count recovery with a median overall survival (OS) time of 9.3 months [86]. In addition, the adenine base editor reduces PD-1 by changing the glycosylated residue in CAR-T cells, which could be used to augment CAR-T therapy [107]. In terms of the structure of CAR, a PD-1-CD28 fusion receptor is able to switch original inhibitory signals into activation signal and enhance cytokine release, proliferation, and cytotoxicity of CAR-T cells [121].

IDO-1 inhibition restricts Tregs in the tumor microenvironment [88]. Indoximod (1-methyl-D-tryptophan, 1MT, NLG-8189) is the most widely studied IDO1 inhibitor, and the combination of IDO1 inhibitors and conventional therapy has shown satisfactory results in several trials.

### Bridging allo-HSCT after CAR T cell therapy can improve patient survival

Brentjens et al. reported four patients who were treated with CAR T cells followed by allo-HSCT [19]. Three patients had no significant complications and remained in MRD-negative complete remission from 3 to 6 months after allo-HSCT. However, one patient who was ineligible for allo-HSCT relapsed 3 months after CAR T cell treatment. Maude et al. reported that three patients in remission were treated with CAR T cells followed by allo-HSCT and remained in remission for 7-12 months after CAR T cell treatmen t[17]. In one study, a total of 14 patients with RR-B-ALL were treated with CD19 CAR T cells. Among the patients, seven were treated with autologous CAR T cells, and seven were treated with cells from donors. Eight patients achieved complete remission, but three of these patients relapsed within 3 months (Zhang C et al., unpublished Data). Notably, we treated a B-ALL patient with central nervous system (CNS) recurrence. After treatment with CAR T cells, the patient remains negative for CNS leukemia. Only one patient died on day 10 [122]. Some researchers have shown that CAR T cell treatment with allo-HSCT may be the best way to treat RR-B cell malignancies. Zhang et al. showed that patients with RR-B cell malignancies showed no sign of relapse at their last follow-up examination if they received CAR T cell treatment followed by allo-HSCT [123]. Another study showed that with a median follow-up period of 28.4 months after allogeneic HCT, the 2-year Kaplan-Meier point estimates of event-free survival (EFS) and OS were 61% and 72%, respectively. The 2-year cumulative incidence of relapse (CIR) was 17% (all with CD19-positive disease at relapse), and nonrelapse mortality (NRM) was 23%. The authors evaluated the effect of allogeneic HCT after CAR T cell therapy on EFS as a time-dependent covariate. In univariate analysis, allogeneic HCT after CAR T cell therapy was associated with longer EFS than not undergoing allogeneic HCT [35]. In a clinical trial performed by Tu SF et al., among the eight patients who underwent HSCT, only two patients relapsed, one at 100 days and one at 226 days post-HSCT. Both patients had experienced secondary relapse and had a relatively high disease burden before CAR T-cell infusion. Patients who underwent HSCT after CAR T cell infusion tended to have better disease-free survival (DFS) and OS than patients who were only observed for follow-up; however, statistical significance was not achieved (P= 0.23, P= 0.20) [124].

Therefore, the best way to treat RR-ALL may be to bridge with allo-HSCT after CAR T cell therapy to improve patient survival [125].

### Dual-target CAR-T cells infusion can prevent antigen escape

Except for CD19, CD22 is widely expressed in B cells and most B-ALL cells as well [126]. So, CD19/CD22 bi-specific CAR-T cells can be used to prevent CD19-negative relapse [91]. Because this paper mainly discusses CD19-positive ALL relapse, there will not be much discussion on dual-target CAR-T cells infusion.

## Conclusion

CD19 CAR T cell therapy is an emerging method of immunotherapy for ALL treatment. However, with the increasing number of trials, CD19^+^ relapse has been noticed and hinders the development of this therapy. Thus, we discussed the mechanism underlying CD19^+^ relapse and found that factors exist during the production of CAR T cells, such as poor-quality T cells, use of murine scFvs, use of the costimulatory domain of 4-1BB, and use of viral vectors. Moreover, aberrant signal transduction pathways can lead to relapse via IL-6/JAK/STAT3 signaling and downregulated DR-5 expression and cGAS-STING signaling, for example. In addition, the roles of immunosuppressive regulatory cells and checkpoints in the BM microenvironment cannot be ignored. We also provided some strategies according to what we found. We believe that analyzing the mechanisms of CD19^+^ relapse can promote progress in CD19 CAR T cell therapy.

## Data Availability

All data generated or analyzed during this study are included in this published article [and its supplementary information files].

## Abbreviations

CAR: Chimeric antigen receptor
ALL: Acute lymphoblastic leukemia
CRS: Cytokine release syndrome
HSCT: Hematopoietic stem cell transplantation
Ph+: Philadelphia chromosome-positive
scFv: Single-chain variable fragment
T_CM_ cells: Central memory T
T_SCM_: T memory stem cell
T_N_: Naïve T cells
T_EM_: Effector memory T cells
T_E_: Effector T cells
TRAF2: Tumor necrosis factor receptor-associated factor 2
ICAM-1: Intracellular adhesion molecule 1
ORR: Overall response rate
CTL: Cytotoxic T lymphocyte
SB: Sleeping beauty
TRAIL: Tumor necrosis factor-related apoptosis-inducing ligand
Treg: Regulatory T cell
CTLA-4: Cytotoxic T-lymphocyte-associated protein 4
MDSC: Myeloid-derived suppressor cell
NKG2D: Natural killer group 2D
TAM: Tumor-associated macrophage
iTregs: Induced Tregs
GSK-3β: Glycogen synthase kinase 3β
NAC: N-acetylcysteine
AICD: Activation-induced cell death
NK: Natural killer cells
AML: Acute myelocytic leukemia
